# Information disclosure in clinical informed consent: “reasonable” patient’s perception of norm in high-context communication culture

**DOI:** 10.1186/1472-6939-15-3

**Published:** 2014-01-10

**Authors:** Muhammad M Hammami, Yussuf Al-Jawarneh, Muhammad B Hammami, Mohammad Al Qadire

**Affiliations:** 1 Clinical Studies and Empirical Ethics Department, King Faisal Specialist Hospital and Research Centre, P O Box # 3354 (MBC 03), Riyadh 11211, Saudi Arabia; 2 Alfaisal University College of Medicine, Riyadh, Saudi Arabia

**Keywords:** Clinical informed consent, Middle East, Information disclosure, Norm perception, Current practice, Gender, Age, High-context communication, Blunting-style coping, Reasonable patient’s standard

## Abstract

**Background:**

The current doctrine of informed consent for clinical care has been developed in cultures characterized by low-context communication and monitoring-style coping. There are scarce empirical data on patients’ norm perception of information disclosure in other cultures.

**Methods:**

We surveyed 470 adults who were planning to undergo or had recently undergone a written informed consent-requiring procedure in a tertiary healthcare hospital in Saudi Arabia. Perceptions of norm and current practice were explored using a 5-point Likert scale (1 = strongly agree with disclosure) and 30 information items in 7 domains: practitioners’ details, benefits, risks, complications’ management, available alternatives, procedure’s description, and post-procedure’s issues.

**Results:**

Respondents’ mean (SD) age was 38.4 (12.5); 50.2% were males, 57.2% had ≥ college education, and 37.9% had undergone a procedure. According to norm perception, strongly agree/agree responses ranged from 98.0% (major benefits) to 50.5% (assistant/trainee’s name). Overall, items related to benefits and post-procedure’s issues were ranked better (more agreeable) than items related to risks and available alternatives. Ranking scores were better in post-procedure respondents for 4 (13.3%) items (p < 0.001 to 0.001) and in males for 8 (26.7%) items (p = 0.008 to <0.001). Older age was associated with better ranking scores for 3 (10.0%) items and worse for one (p < 0.001 to 0.006). According to current practice perception, strongly agree/agree responses ranged from 93.3% (disclosure of procedure’s name) to 13.9% (lead practitioner’s training place), ranking scores were worse for all items compared to norm perception (p < 0.001), and post-procedure status, younger age, and lower educational level were associated with better ranking scores for 15 (50.0%), 12 (40.0%), and 4 (13.3%) items, respectively (p < 0.001 to 0.009).

**Conclusions:**

1) even in an overall high-context communication culture, extensive and more information than is currently disclosed is perceived as norm, 2) the focus of the desired information is closer to benefits and post-procedure’s issues than risks and available alternatives, 3) male, post-procedure, and older patients are in favor of more information disclosure, 4) male, older, and more educated patients may be particularly dissatisfied with current information disclosure. The focus and extent of information disclosure for clinical informed consent may need to be adjusted if a “reasonable” patient’s standard is to be met.

## Background

In addition to its important moral goals [1,2], the informed consent process, if appropriately used, can promote efficient health care through protecting patients from undue risks, enhancing recovery, promoting better pain tolerance, reducing anxiety and depression, encouraging cooperation, increasing satisfaction, building trust, and reducing cost [3,4]. Although the informed consent doctrine is integral to current clinical practice [5], which aims to be individualized and evidence-based, it continues to be primarily a legal and ethical concept that has been developed in Western culture [6,7] and influenced by clinical research atrocities [8].

Despite the similarities between informed consent for clinical care (clinical informed consent) and informed consent for research, there are important differences [9]. In clinical care, patients need and seek care (informed “request”), benefits and risks are better defined, the encounter’s aim is to benefit individual patients rather than patients in general, and practitioners are more formally trained and licensed. Therefore, the focus and extent of information disclosure may be different. Consent for surgery arose in the early 20th century as US courts moved to protect patients from battery and negligence and “informed” consent was first articulated in 1957, when a court called for disclosure of risks to enable patient’s decision-making [10,11]. Interestingly, a forerunner of informed consent was documented in the Eastern Mediterranean region at least since the mid-17th century [12,13]. The current medical practice law in Saudi Arabia is not dissimilar to the corresponding Western and international regulations [14]. The Saudi Arabian Ministry of Health Rules of Implementation for Regulation of the Practice of Medicine and Dentistry (1988) state that “prior to delivering medical treatment or carrying out an operative procedure, the legally competent patient’s consent, be he/she male or female, shall be obtained.”, and “the physician shall provide adequate explanation to the patient or his guardian on the nature of the medical treatment or operative procedure he intends to apply.” However, the Rules do not clarify what “adequate explanation” means. Further, the World Medical Association (WMA) Declaration of Lisbon on the rights of the patient states under Right to information: “Information should be given in a way appropriate to the patient’s culture and in such a way that the patient can understand.” and “The patient has the right not to be informed on his/her explicit request, unless required for the protection of another person’s life” [14].

With the movement to a patient-centered healthcare approach, there has been a shift in information disclosure’s norm from a “reasonable” clinician’s standard to a “reasonable” patient’s standard [2], which can only be defined empirically and is expected to be culture-sensitive. Cultures have been classified into high-context communication cultures (typical for collectivist cultures), where most of the information is either in the physical environment or supposed to be known and little has to be said, and low-contest communication cultures (typical for individualistic cultures), where the mass of information has to be directly communicated [15]. On an Individualism Index Scale, Arab countries have a score of 38 out of 100 compared, for example, to 91 for the United States [16]. It has been suggested that disclosing too much information in a high-context communication culture may have a paradoxical effect of raising suspicion of withholding information and that such culture idiosyncrasies may play a critical role in determining standards of information disclosure in relation to clinical informed consent [6]. Further, clinical procedures are stressful and a person’s internalized cultural norms affect the appraisal process of stressors and the perceived appropriateness of coping responses [17]. A recent review revealed compelling evidence for cultural specificities in coping behavior, with cultural syndromes of collectivism and individualism being the most frequently implicated constructs [18]. Individuals with monitoring coping style cope by gaining as much information as possible, whereas those with blunting coping style cope by avoiding information [3]. Interestingly, there was an interaction between coping style and information disclosure in relation to recovery from surgery [3,19]. Blunting coping style is more common among Asians [18]. Furthermore, it has been argued that there are two types of information to consider in the informed consent process, information that is important in order to give permission and information that is important in order to make an informed choice, and that individuals differ in the kinds of information they want to know/use when making an informed choice [20].

A “reasonable” patient’s standard of information disclosure may have to be shaped by empirical studies on patients’ perceptions in a given culture rather than being based on Western-centric or acontextual assumptions [6,11,21]. There are scarce empirical data to guide clinicians and policy makers on what information a “reasonable” patient likes to be disclosed in the informed consent process, especially in cultures characterized by high-context communication and blunting coping style. The aim of this study was to explore the desired information disclosure in patients who were planning to undergo or who had recently undergone a written informed consent-requiring procedure in a tertiary healthcare center in Saudi Arabia.

## Methods

This cross sectional survey was based on a convenience sample of tertiary care hospital attendees and was conducted in accordance with the ethical principles contained in the Declaration of Helsinki and after approval of the Research Ethics Committee (REC) of the King Faisal Specialist Hospital and Research Center (KFSH&RC). A request of waiver of written informed consent was approved by the REC and all respondents gave verbal consent.

Adult patients who had undergone a medical or surgical procedure that requires a specific written informed consent in the last 6 months or were planning to undergo one within the next 3 months, who were able to understand the purpose and procedures of the study, and who provided verbal informed consent, were eligible to participate. The study was exploratory; sampling method and sample size were convenience-based with the aim to have around 500 evaluable responses. Participants were recruited by research coordinators in the waiting areas of the outpatients’ clinics. Research coordinators identified themselves as such to ensure that respondents would not give answers that they thought might be expected by healthcare professionals. The questionnaire was self-administered in Arabic language with research coordinators’ support as requested by respondents. A research coordinator was available at all times to assist respondents to complete the questionnaire and answer questions regarding the comprehension of the questionnaire. The following demographic data were collected, age, gender, whether the respondent had undergone or was going to undergo a procedure, and educational level (illiterate, primary school, intermediate school, secondary school, college, university).

During the development phase, we wanted to ensure that questionnaire’s items will be understood by respondents as we have intended and that we have covered all pieces of information that patients undergoing such procedures may be interested in. This was iteratively evaluated by means of focused probing in the interview session following completion of the questionnaire. In total 20 respondents were interviewed, 10 during face validity assessment and 10 during pilot testing of the final version (for acceptability, comprehensibility, and 2–3 days stability). We had to add 2 and reward 4 items during face validity assessment phase but none during pilot testing. The results of pilot testing were not included in this report. The questionnaire consists of two parts: one on perception of norm and one on perception of current practice at KFSH&RC. Each part presented participants with a total of 30 items that covered 7 domains of information: involved practitioners’ details (lead practitioner’s name and title, place of training, years of experience, number of similar procedures performed, and success rate; anesthesiologist’s name and title; assistant/trainee’s name), benefits (major, moderate, and minor), risks (major risks, major risks with frequencies, moderate risks, moderate risks with frequencies, minor risks), complications’ management (availability, where, who bear cost), available alternatives (city, country, worldwide), procedure’s description (name, simple, detailed), and post-procedure’s issues (recovery time, feeding, urination and bowel movement, pain/discomfort, special requirements such as for bathing, etc., time to return to work). An English translation (accuracy confirmed by back translation) of the questionnaire and instructions given to participants is available in Additional file 1. Respondents were asked to complete a 5-point Lickert scale (1 = strongly agree, 2 = agree, 3 = neutral, 4 = disagree, 5 = strongly disagree) for each item.

Data were verified by double entry and validity checks were undertaken. The number and percentage of respondents who assigned each of the 5 ranks were calculated for each item. Mean (SD) and median [25%, 75%] ranking scores were also determined for each item. Wilcoxon Signed Ranks test was used to compare perceptions of norm and current practice for each item. Mann–Whitney test was used to compare males to females and respondents who had undergone a procedure (post-procedure respondents) to respondents who were planning to undergo one (pre-procedure respondents). Jonckheere-Terpstra test was used to study the trend in ranking scores among 3 educational subgroups (up to intermediate school, secondary school/college, and university) and four age subgroups: <30 (#140), 30 to 39 (#138), 40 to 49 (#93), and ≥ 50 (# 99). A 2-tailed p value of <0.01 was considered significant. 2-tailed p values are reported. Analyses were conducted using SPSS for Windows software (release 17.0.0, 2008. SPSS Inc., Chicago, ILL, USA).

## Results

Evaluable questionnaire were returned by 470 respondents. Ninety percent of respondents ranked ≥90% of the 30 items, and each of the 30 items was ranked by at least 86% of respondents. 96.8% of respondents were Saudis, 2.5% Non-Saudi Arabs, and 0.7% of other nationalities. Other respondents’ characteristics are summarized in Table 1.

**Table 1:** Characteristics of study participants (no. = 470)

**Age-mean (SD), yr**	38.4 (12.5)
**Gender-no. (%)**	
Male	236 (50.2)
Female	234 (49.8)
**Procedure/surgery-no. (%)**	
Had in previous 6 months	178 (37.9)
Will have within 3 months	292 (62.1)
**Education level-no. (%)**	
Illiterate	15 (3.2)
Primary school	28 (6.0)
Intermediate school	44 (9.4)
Secondary school	113 (24.2)
College	55 (11.8)
University	212 (45.4)

### Norm perception of information disclosure

**Figure 1:**
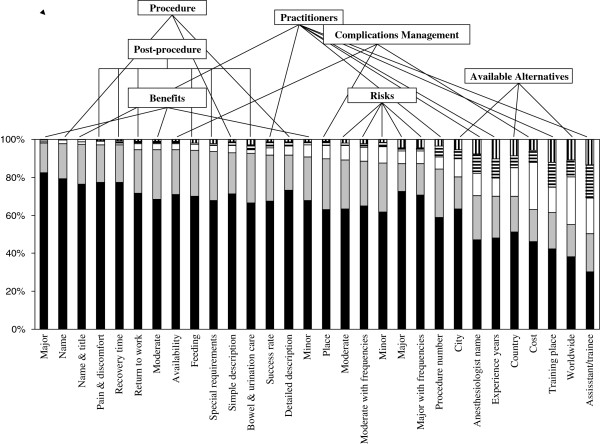
**Patients’ norm perception of information disclosure in clinical informed consent.** Data represent percentage of time each information item was ranked strongly agree (black bars), agree (gray bars), neutral (open bars), disagree (bars with horizontal lines), and strongly disagree (bars with vertical lines). For full description of items, see text and Additional file 1.

Respondents completed a 5-point Likert scale (1 = strongly agree) according to their norm perception of disclosure of information in seven domains (3 to 7 items each). The results are shown in Figure 1. Respondents most frequently agreed with disclosure of major benefits (98.0% agreed/ strongly agreed, mean (SD) score 1.21(0.53)), followed closely by procedure’s name (97.9% agreed/ strongly agreed, mean score 1.23 (0.49)), and lead practitioner’s name and title (97.5% agreed/ strongly agreed, mean score 1.27 (0.54)). They least frequently agreed with disclosure of assistant/trainee’s name (50.5% agreed/ strongly agreed, mean score 2.63 (1.41)) followed by available alternatives worldwide (55.0% agreed/ strongly agreed, mean score 2.37 (1.35)), and lead practitioner’s training place (61.4% agreed/ strongly agreed, mean score 2.33 (1.44)). Further, disclosure of 87% of the 30 items was ranked as strongly agree/agree by 70.0% to 98.0% of respondents. The most disagreement was with disclosure of assistant/ trainee’s name, lead practitioner’s training place, and lead practitioner’s years of experience (30.8%, 25.1%, and 20.5%, respectively, disagreed/strongly disagreed). Overall, there was more agreement with information disclosure in the benefits, procedure, and post-procedure domains than in the risks and available alternatives domains (Figure 1).

### Current practice perception of information disclosure

**Figure 2:**
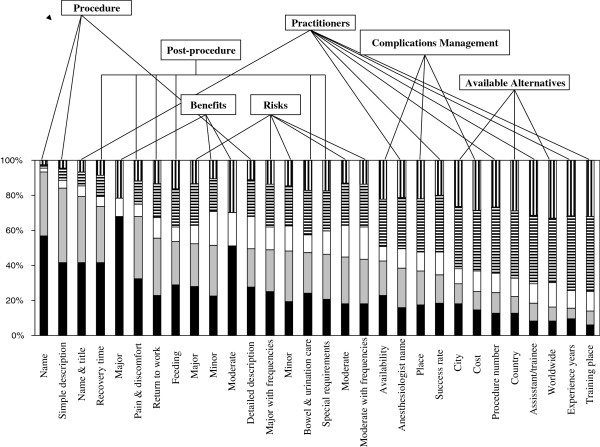
**Patients’ current practice perception of information disclosure in clinical informed consent.** Data represent percentage of time each information item was ranked strongly agree (black bars), agree (gray bars), neutral (open bars), disagree (bars with horizontal lines), and strongly disagree (bars with vertical lines). For full description of items, see text and Additional file 1.

Respondents also completed the same Likert scale according to their perception of information disclosure under current practice. The results are shown in Figure 2. Respondents most frequently agreed with disclosure of procedure’s name (93.3% agreed/ strongly agreed, mean score 1.57 (0.84)), followed by procedure’s simple description (84.2% agreed/ strongly agreed, mean score 1.90 (1.06)), and lead practitioner’s name and title (79.5% agreed/ strongly agreed, mean score 2.00 (1.18)). They least frequently agreed with disclosure of lead practitioner’s training place (13.9% agreed/strongly agreed, mean score 3.87 (1.13)) followed by lead practitioner’s years of experience (15.7% agreed/strongly agreed, mean score 3.81 (1.22)) and available alternatives worldwide (16.3% agreed/ strongly agreed, mean score 3.78 (1.22)). Overall, there was more agreement with information disclosure in the procedure and post-procedure domains than in complications’ management and available alternatives domains (Figure 2).

Ranking was significantly better (more agreeable) according to norm perception than to current practice perception for each of the 30 items (p < 0.001). Further, overall ranking of the risks’ domain was worse according to norm perception compared to current practice perception (Figures 1 and 2).

### Subgroup analysis

**Figure 3:**
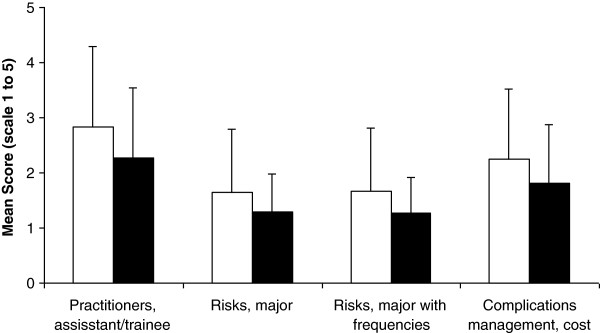
**Comparison of norm perception of information disclosure between pre-procedure (open bars) and post-procedure patients (black bars).** Data represent mean and SD. Only information items with significant differences are included (p value for Mann–Whitney test ranged from <0.001 to 0.001). For full description of items, see text and Additional file 1.

As shown in Figure 3, four (13.3%) items were ranked significantly better according to norm perception by post-procedure respondents compared to pre-procedure respondents: assistant/trainee’s name (2.28 (1.25) vs. 2.84 (1.46), p < 0.001), major risks (1.28 (0.70) vs.1.64 (1.14), p = 0.001), major risks with frequencies (1.28 (0.64) vs. 1.67 (1.14), p < 0.001 ), and costs of complications’ management (1.81 (1.06) vs. 2.25 (1.27), p = 0.001). None of the items was ranked significantly better by pre-procedure respondents. On the other hand, according to current practice perception, 15 (50%) items (in all domains except procedure) were ranked significantly better by post-procedure respondents compared to pre-procedure respondents (p = 0.006 to p < 0.001). None of the 30 items was ranked significantly better by pre-procedure respondents.

**Figure 4:**
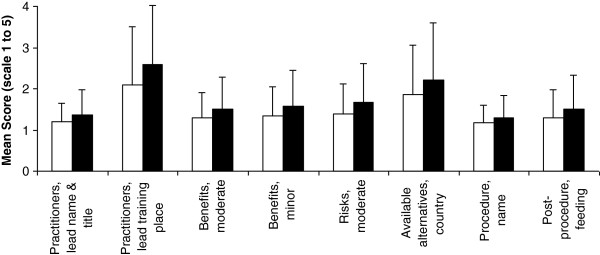
**Comparison of norm perception of information disclosure between males (open bars) and females (black bars).** Data represent mean and SD. Only information items with significant differences are included (p value for Mann–Whitney test ranged from <0.001 to 0.008). For full description of items, see text and Additional file 1.

As shown in Figure 4, eight (26.7%) items were ranked better according to norm perception by males compared to females: lead practitioner’s name and title (1.19 (0.45) vs. 1.36 (0.62), p = 0.001), lead practitioner’s place of training (2.10 (1.40) vs. 2.58 (1.44), p < 0.001), moderate benefits (1.29 (0.62) vs. 1.51 (0.76), p < 0.001), minor benefits (1.34 (0.70) vs. 1.58 (0.88), p = 0.001), moderate risks (1.40 (0.73) vs. 1.67 (0.93), p = 0.001), available alternatives in the country (1.85 (1.19) vs. 2.21 (1.39), p = 0.008), procedure’s name (1.17 (0.44) vs. 1.29 (0.54), p = 0.007), and post-procedure feeding (1.30 (0.68) vs. 1.51 (0.68), p < 0.001). None of the 30 items was ranked significantly better by females according to perception of norm. In contrast, according to current practice perception, 2 items were ranked significantly better by females (anesthesiologist’s name, p = 0.009 and minor benefits, p = 0.001) and one item was ranked better by males (post-procedure recovery time, p = 0.005).

**Figure 5:**
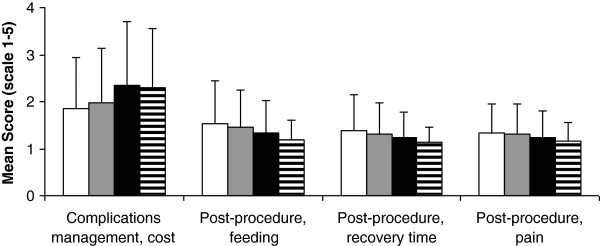
**Comparison of norm perception of information disclosure among five age groups: <30 years (no 140, open bars), 30 to 39 years (no 138, gray bars) 40 to 49 years (no 93, black bars), and ≥ 50 years (no 99, open bars with horizontal lines).** Data represent mean and SD. Only information items with significant differences are included (p value for Jonckheere-Terpstra test ranged from <0.001 to 0.006). For full description of items, see text and Additional file 1.

As shown in Figure 5, according to norm perception, older age was associated with better ranking of 3 (10.0%) items (post-procedure feeding (p < 0.001), post-procedure recovery time (p = 0.002), and post-procedure pain (p = 0.006)) and with worse ranking of one item (complications management cost (p = 0.002)). However, according to current practice perception, older age was associated with worse ranking of 12 (40.0%) items (distributed in all information domains except benefits and risks, p < 0.001 to p = 0.009). Finally, there was no significant association between educational level and ranking scores of any of the items according to perception of norm. However, according to current practice perception, higher educational level was associated with worse ranking of 4 (13.3%) items (major risks, major risks with frequencies, simple description of procedure, and time to return to work, p = 0.001 to 0.008).

## Discussion

Consistent with a patient-centered healthcare approach, there has been a shift in information disclosure’s during the informed consent process to a “reasonable” patient’s standard [2], which is best defined empirically [11,21] and is expected to be culture-sensitive [14]. The main aim of this study was to obtain empirical data on patients’ norm perception of information disclosure in a culture characterized by high-context communication [15] and blunting coping style [18]. Secondary aims were to explore whether norm perception is associated with certain demographics and how it compares to perception of current practice. The strengths of the study include, relatively large sample size, simultaneous examination of perceptions of norm and current practice, surveying actual patients rather than general public, and uniquely addressing an overall high-context communication/blunting coping style culture. We found that: 1) even in such culture, extensive and more information than is currently disclosed is perceived as norm, 2) the focus of the desired information is closer to benefits and post-procedure’s issues than risks and available alternatives, 3) male, post-procedure, and older patients are in favor of more information disclosure, 4) male, older, and more educated patients may be particularly dissatisfied with current information disclosure.

Arab countries, including Saudi Arabia, are characterized by high-context communication culture [15] and blunting coping style [18]; it was hypothesized that in such cultures using “Western” reasonable patient standard of information disclosure may not be appropriate [6]. Our results are not consistent with such hypothesis. Disclosure of 87% of the 30 information items was ranked as strongly agree/agree by 70.0% to 98.0% of our respondents. Our results are more consistent with the results of other studies in other cultures showing, for example, that 84% of pregnant women preferred to have as much information as possible regarding genetic carrier screening [22] and that 17% of patients were not satisfied with the amount of information they received during pre-operative informed consent [23]. Although it is possible that our study sample does not accurately reflect the Saudi culture in general or that the Saudi culture has changed, it is likely that cultural models may not evenly apply to the various communication domains (i.e., medical vs. non-medical).

Although the informed consent doctrine is integral to clinical practice [5] it continues to be primarily an ethical and legal concept [7,10] that was influenced by clinical research atrocities and resulting regulations [8]. It has been argued that in the informed consent process, there are two types of information, information that should be understood and information that only has to be provided in an understandable fashion (doesn’t necessarily need to be understood) [20]. The first type of information is more important from ethical point of view and includes information about risks that are more than minor but not necessarily information about benefits [20]. Further, the arguments in most malpractice litigation cases are focused on adequacy of warnings about risks [7] and clinical research regulations understandably emphasize risks’ disclosure. Interestingly, in contrast to the above considerations, we found that overall, patients are more interested in information items that are related to benefits and post-procedure’ issues than those related to risks.

91.8%, 84.3%, 70.0%, and 61.4% of our respondents, respectively, strongly agreed/agreed that information about lead practitioner success rate, number of procedures performed, years of experience, and place of training should be disclosed during the informed consent process. Further, 70.2% and 50.5%, respectively, strongly agreed/agreed that anesthesiologist and assistant/trainee names should be disclosed. Our results are consistent with a New Zeeland study showing that one of the four most important pieces of information for the patients was related to the operator [24] as well as with some recommendations [25] and laws [26] on performance disclosure. It is of note that US courts have been hesitant to expand the informed consent doctrine to encompass physician-specific variables [27] and that Canadian courts are unlikely to require medical professionals to voluntarily disclose (to non-inquiring patient) their performance data to patients in order to obtain informed consent [7]. Further, 80.3%, 70.0%, and 55.0% of our respondents, respectively, strongly agreed/agreed that information on available alternatives in the city, country, and worldwide should be disclosed. Clinicians may be obligated by law to explain alternative means of treatment and their risks [26].

Compared to pre-procedure respondents, post-procedure respondents ranked the following information items significantly better, assistant/trainee’s name, major risks, major risks with frequencies, and cost of complications’ management; suggesting that undergoing a procedure/consent process might change norm perception of information disclosure toward a wider and more “negative” perspective, which should be taken into account in designing future studies. Information related to common risks and major risks has been identified among the four [24] and five [28] most important pieces of information to patients.

We found that 26.7% of the information items were ranked significantly better according to norm perception by males compared to females. These items appear to be non-specifically distributed among the seven information domains. None of the items was ranked significantly better by females, suggesting that the “reasonable” patient’s standard may be gender specific. A relatively lower interest in information in females may be attributed to lower health self-efficacy level (confidence level in effectively understanding the information, handling the task, and succeeding) and/or to a higher prevalence of blunting coping style, which in turn may be related to a culture dominated by males who are expected to be in control and where females are expected to take a passive role [29]. Further, it has been suggested that, in general, males adopt more problem-focused coping strategies and that females adopt more emotion-focused approaches [30]. Our data seem less consistent with the selectivity model of decision-making, which indicates that females are comprehensive information processors as compared to males who are selective information processors [31]. On the other hand, according to a cost/benefit model of decision-making there is often a compromise between the press for more accuracy and the resistance to personal resources expenditure [32]. Thus it is possible that some information items were less important to females in our study or that they have less available personal resources. We have previously showed gender differences in norm perception of consenting options for posthumous organ donation [33] but not of consenting for retrospective research [34].

We found significantly better ranking of all the information items according to norm perception compared to current practice perception. This is consistent with previous studies showing that 58% of patients were not informed about other possible therapeutic choices during pre-operative informed consent [23], that only 47% of respondents considered informed consent as a means of giving information [35], that 46% of patients believed that the main function of informed consent is to protect hospitals from litigation [36], and that the informed consent process is not satisfactorily applied [37]. Further, in an analysis of informed consent documents used in the management of law back pain by member institutions of the Association of Chiropractic Colleges, the mean number of questions that could be potentially answered with the information provided was only 6.5 out of 20 important questions [38].

The discrepancy between perceived norm and perceived current practice may indicate that current information disclosure does not meet the “reasonable” patient standard. However, other factors should be considered. For example, it was found that carotid endarterectomy patients’ estimates of baseline risk of stroke were significantly different from what they were told [39]. According to fuzzy-trace theory, people extract dual-memory representations of statistical information, coding information gist (qualitative) and the verbatim information; long term memory is mainly supported by reconstruction from gist [39,40]. Further, differences would be expected among “proximal” testing after each major section of the informed consent, “recall” testing at the end of the informed consent process, and “retention” testing several months after [41]. Thus it is possible that the perception of current practice in our study may not be accurate. This is consistent with our observation that according to current practice perception, 50% of the items were ranked significantly better by post-procedure respondents. The success of the informed consent process may be related to professional communication skills and stress on both sides [2] and patient-physician relationship [23], and can be improved by incorporating reader-friendly language, better formatting, and a teach-back portion [42]. A similar difference between perceptions of norm and current practice was found in the same population in relation to consenting options for posthumous organ donation [33] and consenting for retrospective research [34]. The significant difference in ranking scores between perceptions of norm and current practice together with the observed associations with age, gender, and educational level, suggest that there may be some degree of patients’ dissatisfaction with current information disclosure, especially in male, older, and more educated patients.

### Study limitations

The most important considerations in the interpretation of our findings are that the study was based on convenience sampling and was performed in a single tertiary healthcare institution in a major metropolitan city; the results may not be generalizable to the general public. Further, the study sample overrepresented individuals with higher educational level. However, it is of note that the institution is a governmental referral center for the entire country and that educational level was not associated with ranking scores of any information item according to norm perception. Further, our study only addressed written informed consent-requiring procedures; the results may not apply to other healthcare situations with lower risks and/or simpler decisions.

## Conclusions

In the setting of a tertiary care hospital in Saudi Arabia, we found that: 1) Even in an overall high-context communication, blunting coping style culture, extensive and more information than is currently disclosed in the informed consent process is perceived as norm, suggesting that cultural models may not evenly apply to the various communication domains (i.e., medical vs. non-medical). 2) The focus of the desired information is closer to benefits and post-procedure’s issues than risks and available alternatives, suggesting some discrepancy between prevailing laws and ethical concepts and what patients’ desires. 3) Norm perception of information disclosure may be age and gender dependent. 4) Male, older, and more educated patients may be particularly dissatisfied with current information disclosure. The focus and extent of information disclosure may need to be adjusted if a “reasonable” patient’s standard is to be met. The observed differences in perception of information disclosure in relation to time of procedure should be taken into account in designing future studies.

## Competing interests

The authors declare that they have no competing interests.

## Authors’ contributions

MMH designed the study, performed statistical analysis, and wrote the manuscript. YJ participated in data collection. MBH participated in statistical analysis and literature review and co-wrote the manuscript. MAQ participated in study design and literature review. All authors read and approved the final manuscript.

## Pre-publication history

The pre-publication history for this paper can be accessed here:

http://www.biomedcentral.com/1472-6939/15/3/prepub

## Supplementary Material

Additional file 1 **Study Questionnaire.** An English translation of the questionnaire and instructions given to participants. Click here for file
