# Supplementary material for: Information disclosure in clinical informed consent: “reasonable” patient’s perception of norm in high-context communication culture
Source: BMC Med Ethics. 2014 Jan 10;15:3. doi: 10.1186/1472-6939-15-3 (PMC3930349; doi:10.1186/1472-6939-15-3)
Supplement: Additional file 1 — Study Questionnaire. An English translation of the questionnaire and instructions given to participants. [file 1472-6939-15-3-S1.doc]

**PART I:** Please indicate how much you agree or disagree that the following pieces of information are **currently** **provided** to patients when they consent to undergo surgical/medical procedures at the King Faisal Specialist Hospital & Research Center. There are 30 pieces of information divided into 7 sections. For each piece of information, please choose one of the following responses: strongly agree, agree, neutral, disagree, and strongly disagree. Thank you.

| Strongly  Disagree | Disagree | Neutral | Agree | Strongly Agree |  |
| --- | --- | --- | --- | --- | --- |
| **Responsible practitioners** | | | | | |
|  |  |  |  |  | Name and title of lead practitioner |
|  |  |  |  |  | Place of training of lead practitioner |
|  |  |  |  |  | Years of experience of lead practitioner |
|  |  |  |  |  | Number of similar procedures performed by lead practitioner |
|  |  |  |  |  | Success rate of lead practitioner |
|  |  |  |  |  | Name and title of anesthesiologist |
|  |  |  |  |  | Name of Assistants/Trainees |
| **Benefits of recommended procedure** | | | | | |
|  |  |  |  |  | Major benefits |
|  |  |  |  |  | Moderate benefits |
|  |  |  |  |  | Minor benefits |
| **Risks of recommended procedure** | | | | | |
|  |  |  |  |  | Major risks |
|  |  |  |  |  | Major risks with their frequencies |
|  |  |  |  |  | Moderate risks |
|  |  |  |  |  | Moderate risks with their frequencies |
|  |  |  |  |  | Minor risks |
| **Management of complications** | | | | | |
|  |  |  |  |  | Whether available or not |
|  |  |  |  |  | If available, where? |
|  |  |  |  |  | If available, who does bear cost? |
| **Available alternatives to recommended procedure** | | | | | |
|  |  |  |  |  | Available alternatives in Riyadh |
|  |  |  |  |  | Available alternatives in KSA |
|  |  |  |  |  | Available alternatives Worldwide |
| **Description of recommended procedure** | | | | | |
|  | | | | | Name only |
|  |  |  |  |  | Simple description (only major components) |
|  |  |  |  |  | Detailed description (major and minor components) |
| **After procedure issues** | | | | | |
|  |  |  |  |  | Recovery time |
|  |  |  |  |  | Feeding |
|  |  |  |  |  | Urination and bowel movement |
|  |  |  |  |  | Pain /discomfort |
|  |  |  |  |  | Special requirements (bathing, etc.) |
|  |  |  |  |  | Time to return to work |

**PART II:** Please indicate how much you agree or disagree that the following pieces of information **should be provided** to patients when they consent to undergo surgical/medical procedures. There are 30 pieces of information divided into 7 sections. For each piece of information, please choose one of the following responses: strongly agree, agree, neutral, disagree, and strongly disagree. Thank you.

| Strongly  Disagree | Disagree | Neutral | Agree | Strongly Agree |  |
| --- | --- | --- | --- | --- | --- |
| **Responsible practitioners** | | | | | |
|  |  |  |  |  | Name and title of lead practitioner |
|  |  |  |  |  | Place of training of lead practitioner |
|  |  |  |  |  | Years of experience of lead practitioner |
|  |  |  |  |  | Number of similar procedures performed by lead practitioner |
|  |  |  |  |  | Success rate of lead practitioner |
|  |  |  |  |  | Name and title of anesthesiologist |
|  |  |  |  |  | Name of Assistants/Trainees |
| **Benefits of recommended procedure** | | | | | |
|  |  |  |  |  | Major benefits |
|  |  |  |  |  | Moderate benefits |
|  |  |  |  |  | Minor benefits |
| **Risks of recommended procedure** | | | | | |
|  |  |  |  |  | Major risks |
|  |  |  |  |  | Major risks with their frequencies |
|  |  |  |  |  | Moderate risks |
|  |  |  |  |  | Moderate risks with their frequencies |
|  |  |  |  |  | Minor risks |
| **Management of complications** | | | | | |
|  |  |  |  |  | Whether available or not |
|  |  |  |  |  | If available, where? |
|  |  |  |  |  | If available, who does bear cost? |
| **Available alternatives to recommended procedure** | | | | | |
|  |  |  |  |  | Available alternatives in Riyadh |
|  |  |  |  |  | Available alternatives in KSA |
|  |  |  |  |  | Available alternatives Worldwide |
| **Description of recommended procedure** | | | | | |
|  | | | | | Name only |
|  |  |  |  |  | Simple description (only major components) |
|  |  |  |  |  | Detailed description (major and minor components) |
| **After procedure issues** | | | | | |
|  |  |  |  |  | Recovery time |
|  |  |  |  |  | Feeding |
|  |  |  |  |  | Urination and bowel movement |
|  |  |  |  |  | Pain /discomfort |
|  |  |  |  |  | Special requirements (bathing, etc.) |
|  |  |  |  |  | Time to return to work |
